# Atomic-Scale Characterization of Slip Deformation and Nanometric Machinability of Single-Crystal 6H-SiC

**DOI:** 10.1186/s11671-019-3123-7

**Published:** 2019-09-10

**Authors:** Binbin Meng, Dandan Yuan, Shaolin Xu

**Affiliations:** 1 Department of Mechanical and Energy Engineering, Southern University of Science and Technology, Shenzhen, 518055 People’s Republic of China; 2 School of Power and Mechanical Engineering, Wuhan University, Wuhan, 430000 People’s Republic of China

**Keywords:** 6H-SiC, Anisotropy, Deformation, Machinability

## Abstract

As an important third-generation semiconductor material, the micro-deformation and removal mechanism of 6H-SiC at the atomic scale are vital for obtaining ultra-smooth and damage-free surface with atomic steps. Due to the difficulties in directly observing the surface/subsurface of nanomachining region by current experimental means, molecular dynamics method is used to study the atomic-scale details in nanomachining process, such as dislocation slip motion, phase transition, and material separation mechanism. The influence of crystallography-induced anisotropy on the slip deformation and nanometric machinability of 6H-SiC is emphatically investigated. This study contributes significantly to the understanding of micro-deformation and nanomachining process of 6H-SiC.

## Introduction

As the third generation semiconductor material with wide bandgap, SiC has the characteristics of high breakdown field, high radiation tolerance, high velocity of carrier saturation, fast thermal conductivity, small dielectric constant, and steady chemical properties, so it has wide applications in the fields of high temperature, high frequency, high power, anti-radiation, and short-wavelength optoelectronic devices and optoelectronic integration [1].

The most widely used crystals of SiC are 3C, 4H, and 6H. Processing methods such as grinding/lapping/polishing are still the main methods during the machining of single-crystal SiC. However, the hardness ratio between diamond and SiC is close to 2:1 (the processing depth < 50 nm)), which is much lower than the recommended value of 5:1 for the machining process [2]. Severe wear of cutting tool and subsurface damage directly influence the quality of wafer. To address these issues, a large amount of work has been done to understand the removal behavior of SiC at the nanoscale. The removal mechanism of 3C-SiC and influencing of the processing factors have been thoroughly studied, such as the plastic deformation mechanism during the cutting process [3–7], tool wear [8], friction behavior [9], and anisotropy of 3C-SiC [10] and influence of cutting temperatures [11].

6H-SiC has a more complex ABCACB stack structure. Although the removal mechanism of 6H-SiC in SPDT (single point diamond turning) processing (such as the influence of tool rake angle on the material removal process [12] and brittle-ductile transition [13]) is studied, the research is obviously less than 3C-SiC. The bandgap of 6H-SiC (3 eV) is obviously higher than that of 3C-SiC (2.3 eV). At present, the technological level of 6H-SiC rod growth process is much higher than that of 3C-SiC. 6H-SiC is far more used in industrial applications than 3C-SiC. Corresponding devices have been applied in high frequency, high power, and high-temperature fields, such as Schottky rectifier, thyratron, and power MOSFET (Metal-Oxide-Semiconductor Field-Effect Transistor). To enhance the machined surface/subsurface quality of 6H-SiC, the most efficient and effective means at present is to find an appropriate combination of crystal plane (machining surface)/crystal orientation (machining direction) that is more suitable for the process of 6H-SiC.

Scratch experiment and simulations are some of the most common and effective methods to explore the removal behavior [14, 15]. The research on material removal mechanism during the scratching process has a great guiding significance for the actual abrasive machining process. The molecular dynamics method can provide damage formation and removal process at the nanoscale. Therefore, the molecular dynamics scratching simulation was used to analyze the removal behavior of 6H-SiC under the influence of crystallography-induced anisotropy.

## Methodology

**Fig. 1:**
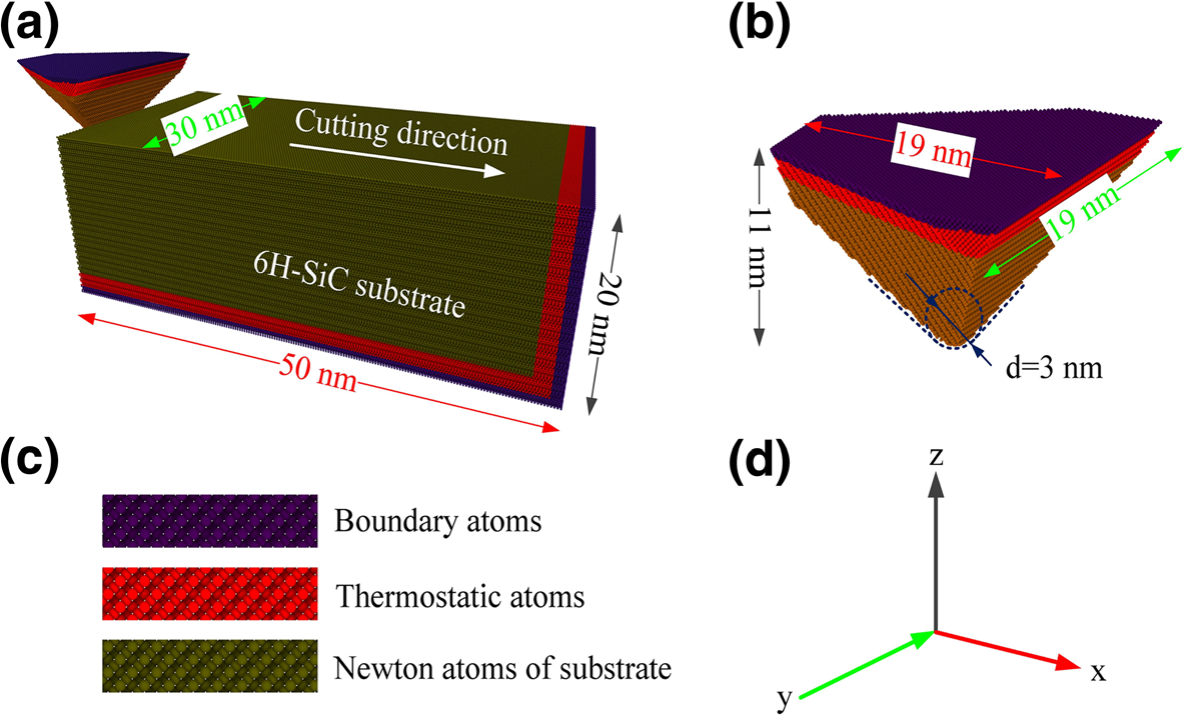
**a** MD model of nanoscratching simulation. **b** The morphology of the tool. **c** Structure of model. **d** Axis direction

**Fig. 2:**
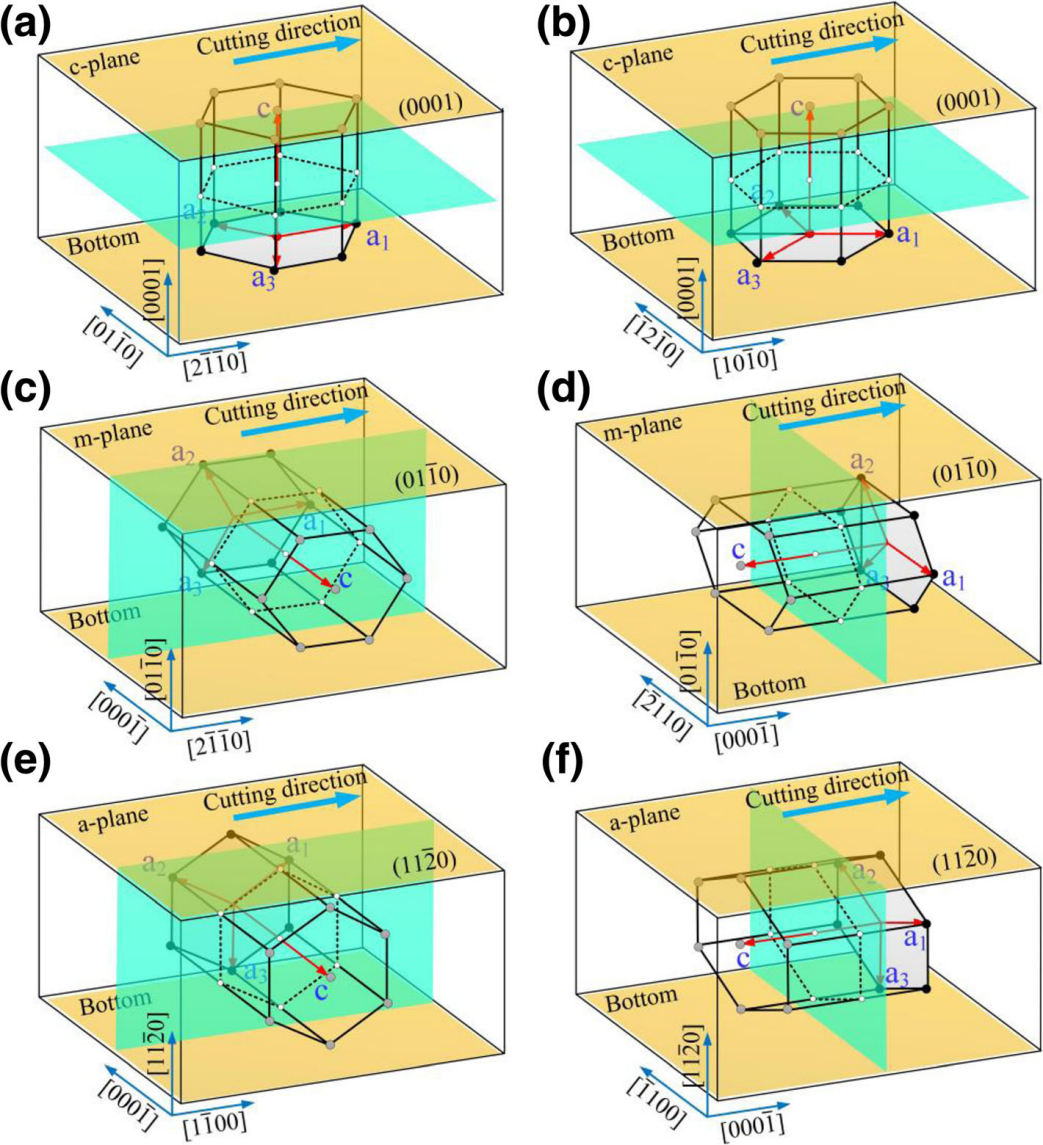
Schematic diagram of scratching process, where **a**–**f** are the corresponding process modes $(0001)/\left[2\overline{1}\overline{1}0\right]$, $(0001)/\left[10\overline{1}0\right]$, $\left(01\overline{1}0\right)/\left[2\overline{1}\overline{1}0\right]$, $\left(01\overline{1}0\right)/\left[0001\right]$, $\left(11\overline{2}0\right)/\left[1\overline{1}00\right]$, and $\left(11\overline{2}0\right)/\left[0001\right]$, respectively

The cutting simulations in this paper were completed using large-scale atomic/molecular massively parallel simulator (LAMMPS) [16]. OIVTO [17] and diamond structure identification method [18] were used for model visualization and defect identification in this study. The implementations of workpiece and tool modeling were dependent on LAMMPS without the aid of other software. As shown in Fig. 1a–c, both the workpiece and the tool were set as deformable bodies, and the wear behavior was investigated during the simulation. The tool and workpiece model were divided into three parts: a boundary atomic layer, a thermostatic atomic layer, and a Newtonian atoms zone. In order to keep the workpiece remain in the initial position, the boundary layer atoms in the bottom and right end of workpiece were fixed. Atoms in thermostat layer and Newtonian layer follow Newton’s second law. A periodic boundary was applied along *y* direction. Before the scratching simulation, the models were relaxed by NVE ensemble with Berendsen thermostat method. A 50 ps relaxation period was applied before the scratching simulations to obtain a steady energy state. The abrasive shape is a spherical crown triangular pyramid with the edge-to-edge angle of 90°. As shown in Fig. 2, the common crystal planes (a-plane (basal plane), m-plane (prismatic plane), and c-plane) for 6H-SiC were selected as the machining surfaces. Considering the symmetry of the structure, the following plane/orientation combinations were selected as the machining surface/machining direction: $(0001)/\left[2\overline{1}\overline{1}0\right]$, $(0001)/\left[10\overline{1}0\right]$, $\left(01\overline{1}0\right)/\left[2\overline{1}\overline{1}0\right]$, $\left(01\overline{1}0\right)/\left[0001\right]$, $\left(11\overline{2}0\right)/\left[1\overline{1}00\right]$, and $\left(11\overline{2}0\right)/\left[0001\right]$. The processing parameters of the simulation are shown in Table 1. Prior to the scratching simulation, the abrasives were placed on the left side of the workpiece, and the abrasive tip is 50 Å below the upper surface of the workpiece. The closest distance between the abrasives and the workpiece is 30 Å which is far from the cut-off range of interaction potential. The abrasive moves from the free end of the workpiece in the positive direction of *x*-axis and complete the scratching process.

**Table 1:** Process variables used in the MD simulation

Details	Parameters
Size of the workpiece	50 nm× 20 nm× 30 nm
Undeformed chip thickness (*d*)	5.0 nm
Tip radius	3.0 nm
Cutting rake angle	− 25°
Equilibration temperature	300 K
Cutting velocity	50 ms^−1^
Timestep	1 fs
Combinations	machining surface/machining direction
Case 1	$(0001)/\left[2\overline{1}\overline{1}0\right]$
Case 2	$(0001)/\left[10\overline{1}0\right]$
Case 3	$\left(01\overline{1}0\right)/\left[2\overline{1}\overline{1}0\right]$
Case 4	$\left(01\overline{1}0\right)/\left[0001\right]$
Case 5	$\left(11\overline{2}0\right)/\left[1\overline{1}00\right]$
Case 6	$\left(11\overline{2}0\right)/\left[0001\right]$

The atomic potential function plays a crucial role in the accuracy and reliability of molecular dynamics simulation. According to the previous tests and simulations for the mechanical properties and removal mechanisms of single-crystal SiC, the analytical bond order potential (ABOP) function proposed by Erhart and Albe [19] is more suitable for interactions for silicon and carbon. The parameters used in the potential function are shown in Table 2 [19]. Instead of the Tersoff potential energy function [6], the ABOP potential function is used to define the Si-Si, C-C, and Si-C interactions in and among the tool and workpiece during the processing [2, 7, 8, 10, 11].

**Table 2:** Potential function parameters used in this study [8]

Details	Si-Si	C-C	Si-C
*D*_*o*_ (eV)	3.24	6	4.36
*r*_*o*_ (Å)	2.232	1.4276	1.79
*S*	1.842	2.167	1.847
*β* (Å^-1^)	1.4761	2.0099	1.6991
*γ*	0.114354	0.11233	0.011877
*c*	2.00494	181.910	273987
*d*	0.81472	6.28433	180.314
h	0.259	0.5556	0.68
*2 μ* (Å^−1^)	0	0	0
*R* (Å)	2.82	2	2.4
*D* (Å)	0.14	0.15	0.2

## Results and Discussion

### Nanometric Machinability Analysis

**Fig. 3:**
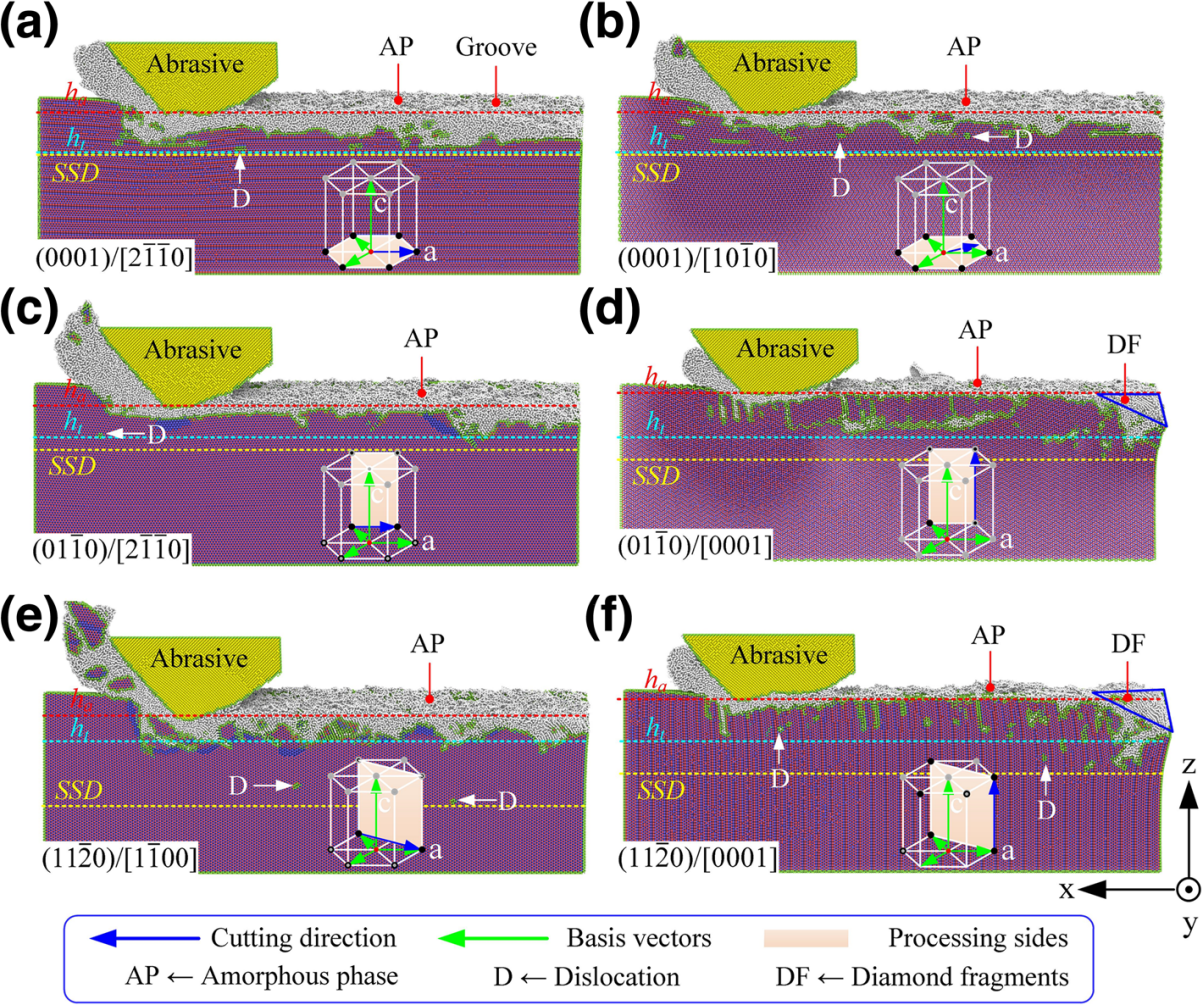
Topography of machined surface under different crystal plane/orientation conditions, where **a**–**f** are the corresponding process modes $(0001)/\left[2\overline{1}\overline{1}0\right]$, $(0001)/\left[10\overline{1}0\right]$, $\left(01\overline{1}0\right)/\left[2\overline{1}\overline{1}0\right]$, $\left(01\overline{1}0\right)/\left[0001\right]$, $\left(11\overline{2}0\right)/\left[1\overline{1}00\right]$, and $\left(11\overline{2}0\right)/\left[0001\right]$, respectively

**Fig. 4:**
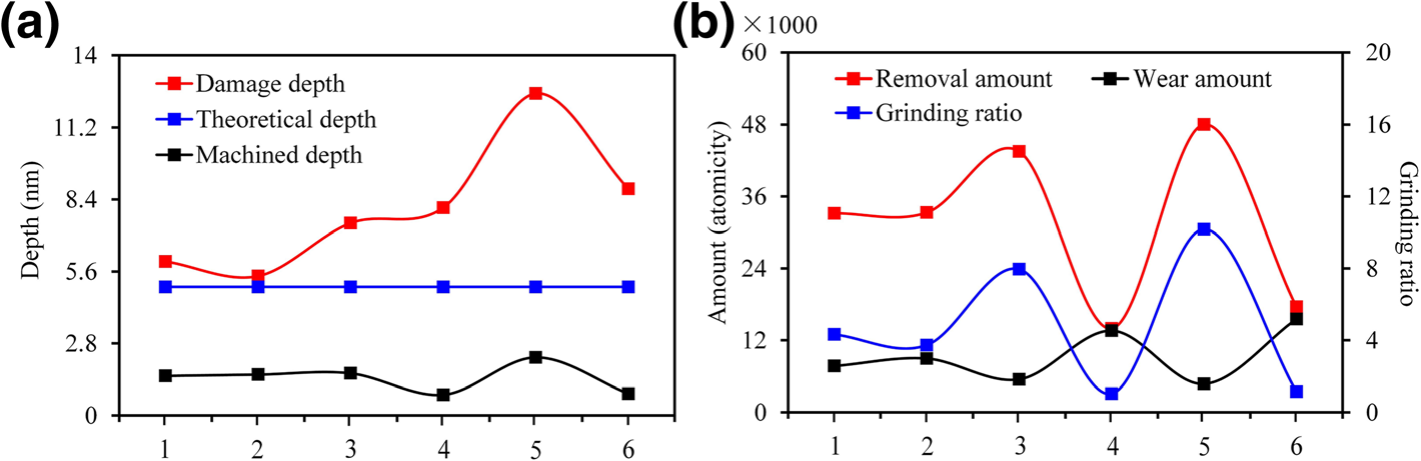
Nanometric machinability under different crystal plane/orientation conditions, **a** the influence of anisotropy on machined depth and damage depth, **b** the influence of anisotropy on removal amount, wear amount and grinding ratio. 1, 2, 3, 4, 5, and 6 are the corresponding process methods $(0001)/\left[2\overline{1}\overline{1}0\right]$, $(0001)/\left[10\overline{1}0\right]$, $\left(01\overline{1}0\right)/\left[2\overline{1}\overline{1}0\right]$, $\left(01\overline{1}0\right)/\left[0001\right]$, $\left(11\overline{2}0\right)/\left[1\overline{1}00\right]$, and $\left(11\overline{2}0\right)/\left[0001\right]$, respectively. The damage layer depth refers to the maximum depth of the internal defects of the crystal caused by the scratching. The theoretical depth refers to the preset depth before processing. The machined depth refers to the residual depth after the scratching. The amount of removal refers to the number of atoms from which the workpiece material is removed. The amount of wear refers to the difference in the number of atoms of the abrasive grains before and after the scratching

As shown in Figs. 3 and 4, the anisotropy of 6H-SiC had a significant impact on the nanometric machinability (machined depth, removal mode, removal amount, and subsurface damage depth (SSD), etc.). According to the relationship between the machining surface/machining direction and basal plane/*c*-axis (see Fig. 3), the material processing modes can be divided into three categories: (i) the basal plane is selected as the machining surface, (ii) the basal plane is perpendicular to the machining surface and *c*-axis is perpendicular to the machining direction, and (iii) the *c*-axis is parallel to the machining direction.

(i) As shown in Fig. 3a, b, the nanometric machinability of 6H-SiC was extremely similar when the processing mode was selected as $(0001)/\left[2\overline{1}\overline{1}0\right]$ and $(0001)/\left[10\overline{1}0\right]$. However, the removal process of 6H-SiC was more inclined towards brittle mode when the processing mode was selected as $(0001)/\left[10\overline{1}0\right]$. (ii) As shown in Fig. 3c, e, when the processing modes were selected as $\left(01\overline{1}0\right)/\left[2\overline{1}\overline{1}0\right]$ and $\left(11\overline{2}0\right)/\left[1\overline{1}00\right]$, the machined depth and material removal amount were significantly higher than those in the other processing methods. As shown in Fig. 4, under the same theoretical processing depth (*h*_*t*_ = 5.0 nm), the material removal rate in the processing mode $\left(11\overline{2}0\right)/\left[1\overline{1}00\right]$ was 3.4 times as much as that in the processing mode $\left(01\overline{1}0\right)/\left[0001\right]$. The material processing ratio (material removal amount/tool wear) was 10.1, but the SSD under this processing parameter was also much higher than that under other processing conditions, reaching 2.3 times of $(0001)/\left[10\overline{1}0\right]$. There were a large number of nanocrystallites on the machined surfaces and chips. A large number of brittle failures occurred under this condition. The processing mode $\left(11\overline{2}0\right)/\left[1\overline{1}00\right]$ is only suitable for nanogroove processing which requires high processing efficiency but is insensitive to the subsurface damage. The material removal rate of $\left(01\overline{1}0\right)/\left[2\overline{1}\overline{1}0\right]$ was similar to that of $\left(11\overline{2}0\right)/\left[1\overline{1}00\right]$ but the SSD was only 50% of the latter. Also, the thickness of the amorphous layer on the machined surface was much lower than that of $(0001)/\left[2\overline{1}\overline{1}0\right]$ and $(0001)/\left[10\overline{1}0\right]$. The subsurface phase distribution was uniform, and the mechanical properties of the whole groove were better than those in the other processing methods. Therefore, the processing mode $\left(01\overline{1}0\right)/\left[0001\right]$ is the best choice for the processing of micro-nano grooves on 6H-SiC surface with high efficiency, high precision, and low SSD. (iii) As shown in Figs. 3d, f, when the machining direction was parallel to *c*-axis, the tip was severely worn during the initial processing stage. Although the machined depth and material removal rate were much lower than those in the other processing methods and the material processing ratio was only about 1.0, the SSD of $\left(01\overline{1}0\right)/\left[0001\right]$ and $\left(11\overline{2}0\right)/\left[0001\right]$ processing modes were more than that of $\left(01\overline{1}0\right)/\left[2\overline{1}\overline{1}0\right]$. Hence, it is not recommended to employ $\left(01\overline{1}0\right)/\left[0001\right]$ and $\left(11\overline{2}0\right)/\left[0001\right]$ in the processing of micro-nano grooves on the surface of 6H-SiC. However, these modes demonstrate an excellent wear resistance; therefore, they are apt for the rake face of the single-crystal SiC cutting tool, which has broad prospects in the field of ultra-precision processing of ferrous metals.

### Analysis of Lip Motion and Subsurface Damage Distribution

#### Schmid Factors Distribution in the Scratching Process Based on a Triangular Pyramid Tip

**Fig. 5:**
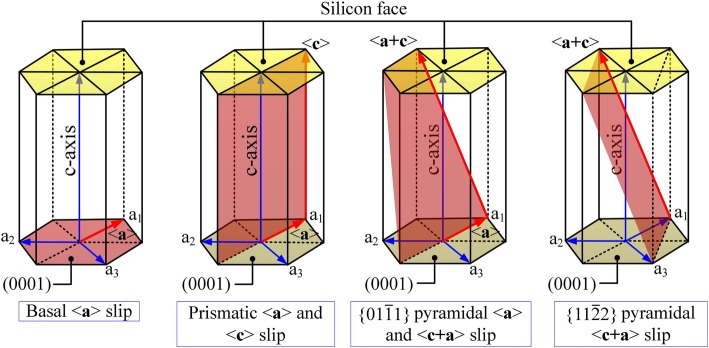
Slip systems of hexagonal system

The common slip systems of hexagonal crystal system (see Fig. 4,) primarily include basal slip, prismatic slip, and pyramidal slip. Slip resistance is related to the generalized stacking fault energy (GSF) and d*E*_GSF_/d*x* vs. (*x*/***b***) of the slip systems. Slip motion would occur on the densest plane and along the shortest direction [10]. The preferential slip systems in the basal slip are basal slip (shuffle sets)/<$1\overline{1}00$> and basal slip (shuffle sets)/<$11\overline{2}0$> [20]. As the former lacks in an intermediate energy minimum, the latter takes precedence in the simulations and experiments [20]. Therefore, the slip motions considered in this work are as follows: basal slip (shuffle sets)/<$11\overline{2}0$> and prismatic <a> slip and <c> slip. Fig. 5.

The shear stress component on the glide plane *τ*_ss_ can be calculated by the relation:

1 $$ {\tau}_{\mathrm{ss}}={\sigma}_{\mathrm{cont}}\mathit{\cos}<\overset{\rightharpoonup }{f},\overset{\rightharpoonup }{n_g}>\cos <\overset{\rightharpoonup }{f},\overset{\rightharpoonup }{t_g}>={\sigma}_{\mathrm{cont}}\bullet m $$

where *σ*_cont_ is the contact stress, $\overset{\rightharpoonup }{f}$ is loading direction, $\overset{\rightharpoonup }{n_g}$ and $\overset{\rightharpoonup }{t_g}$ are the slip plane normal and slip direction in the global Cartesian coordinate system, and *m* is the Schmid factor. The global coordinate system was fixed and the local coordinate system rotated with the direction of the crystal. The *x*-axis of the local coordinate system was parallel to the basic vector $\overset{\rightharpoonup }{a_1}$ while the *z*-axis was parallel with the basic vector $\overset{\rightharpoonup }{c}$.

During the scratching process, the primary contact face of the triangular pyramid tip was the rake face, but when the tool got severely worn, the main contact face transited to the compound of the rake face and the tip-top. The corresponding loading direction $\overset{\rightharpoonup }{f}$ is presented as

2 $$ \left\{\begin{array}{c}\overset{\rightharpoonup }{f_1}=\left({f}_{1x},{f}_{1y},{f}_{1z}\right)\kern8em \mathrm{rake}\ \mathrm{face}\ \\ {}\overset{\rightharpoonup }{f_2}=\left({f}_{2x},{f}_{2y},{f}_{2z}\right)\kern9.75em \mathrm{tip}\ \mathrm{top}\end{array}\right. $$

where $\overset{\rightharpoonup }{f_1}=\left(1,0,-\sqrt{2}\right)$, $\overset{\rightharpoonup }{f_2}=\left(0,0,-1\right)$ in the global coordinate system.

As a hexagonal crystal system, the slip system of 6H-SiC can be expressed as {*h k i l*}/ < *u v t w*>. The slip plane normal $\overset{\rightharpoonup }{n_c}$ and slip direction $\overset{\rightharpoonup }{t_c}$ in the local Cartesian coordinate system are shown as

3 $$ \overset{\rightharpoonup }{n_c}=\left(\frac{3}{2}h,\frac{\sqrt{3}}{2}\left(h+2k\right),\frac{3 la}{2c}\right) $$

4 $$ \overset{\rightharpoonup }{t_c}=\left(u-\frac{1}{2}\left(v+t\right),\frac{\sqrt{3}}{2}\left(v-t\right),\frac{c}{a}w\right) $$

The slip plane normal $\overset{\rightharpoonup }{n_g}$ and slip direction $\overset{\rightharpoonup }{t_g}$ in the global Cartesian coordinate system are shown as

4 $$ \overset{\rightharpoonup }{n_g}=T\bullet \overset{\rightharpoonup }{n_c} $$

5 $$ \overset{\rightharpoonup }{t_g}=T\overset{\rightharpoonup }{\bullet {t}_c} $$

where the rotation matrix form from the global coordinate system to the local coordinate system and the corresponding rotation angles are shown in Table 3

**Table 3:** Euler angles under different processing conditions (°)

	Case 1	Case 2	Case 3	Case 4	Case 5	Case 6
*T*	*R*_*z*_ *R*_*y*_ *R*_*x*_	*R*_*z*_ *R*_*y*_ *R*_*x*_	*R*_*x*_ *R*_*y*_ *R*_*z*_	*R*_*x*_ *R*_*y*_ *R*_*z*_	*R*_*x*_ *R*_*z*_ *R*_*y*_	*R*_*x*_ *R*_*y*_ *R*_*z*_
*ρ*	0	0	90	90	90	90
*θ*	0	0	0	− 90	0	-90
*δ*	0	-30	0	0	30	30

Note: *T* is the rotation matrix, *ρ* is the rotation angle around the *x*-axis, *θ* is the rotation angle around the *y*-axis, and *δ* is the rotation angle around the *z*-axis. The rotation matrices *R*_*x*_, *R*_*y*_, and *R*_*z*_ of the corresponding coordinate axis are, respectively, $$ {R}_x=\left[\begin{array}{ccc}1& 0& 0\\ {}0& \cos \rho & -\sin \rho \\ {}0& \sin \rho & \cos \rho \end{array}\right],{R}_y=\left[\begin{array}{ccc}\cos \theta & 0& \sin \theta \\ {}0& 1& 0\\ {}-\sin \theta & 0& \cos \theta \end{array}\right],{R}_z=\left[\begin{array}{ccc}\cos \delta & -\sin \delta & 0\\ {}\sin \delta & \cos \delta & 0\\ {}0& 0& 1\end{array}\right] $$

According to formulas (1)–(5), the Schmid factors of the corresponding slip systems when the loading directions were $\overset{\rightharpoonup }{f_1}$ and $\overset{\rightharpoonup }{f_2}$ are shown in Table 4. (i) The c/a value of 6H-SiC reached 4.901, which is much larger than 1.633. Therefore, the critical shear stress required for the basal slip was lower than that for prismatic slip system. When the machined surface was a basal plane, the coefficient of basal slip system was higher than that of the other slip systems. Hence, when the processing modes were selected as $(0001)/\left[2\overline{1}\overline{1}0\right]$ and $(0001)/\left[10\overline{1}0\right]$, the basal <a> slip motion took place first. (ii) When the processing modes were selected as $\left(01\overline{1}0\right)/\left[2\overline{1}\overline{1}0\right]$ and $\left(11\overline{2}0\right)/\left[1\overline{1}00\right]$, only considering the loading directions $\overset{\rightharpoonup }{f_1}$ and $\overset{\rightharpoonup }{f_2}$, it was theoretically impossible for the basal slip to occur, and the prismatic slip movement took precedence. (iii) When the processing modes were selected as $\left(01\overline{1}0\right)/\left[0001\right]$ and $\left(11\overline{2}0\right)/\left[0001\right]$, as shown in Fig. 4, the tip-top was seriously worn during the initial processing stage and $\overset{\rightharpoonup }{f_2}$ played a crucial role in the scratching process. Therefore, the slip motion would occur in the prismatic slip system symmetrically distributed with YOZ plane in the global coordinates.

**Table 4:**
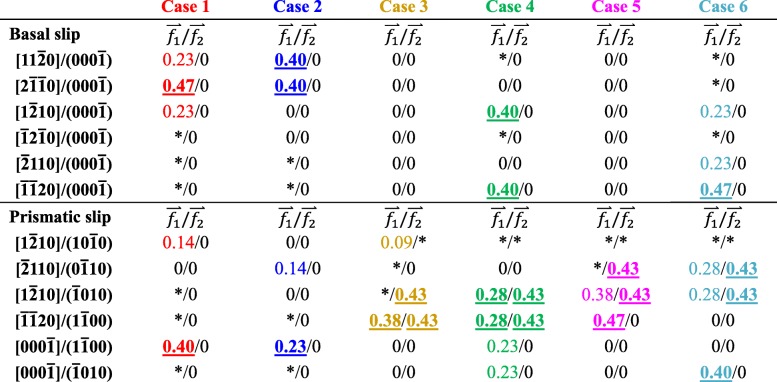
Schmid factors in the MD simulation

Note: Case 1–case 6 correspond to $(0001)/\left[2\overline{1}\overline{1}0\right]$, $(0001)/\left[10\overline{1}0\right]$, $\left(01\overline{1}0\right)/\left[2\overline{1}\overline{1}0\right]$, $\left(01\overline{1}0\right)/\left[0001\right]$, $\left(11\overline{2}0\right)/\left[1\overline{1}00\right]$, and $\left(11\overline{2}0\right)/\left[0001\right]$, respectively. The symbol * means that this slip system will not occur under this loading mode. The font marked with color indicates that the slip system may occur, and the numeric bolding and underlining indicate that the slip system would occur preferentially

#### Surface/Subsurface Damage Distribution

**Fig. 6:**
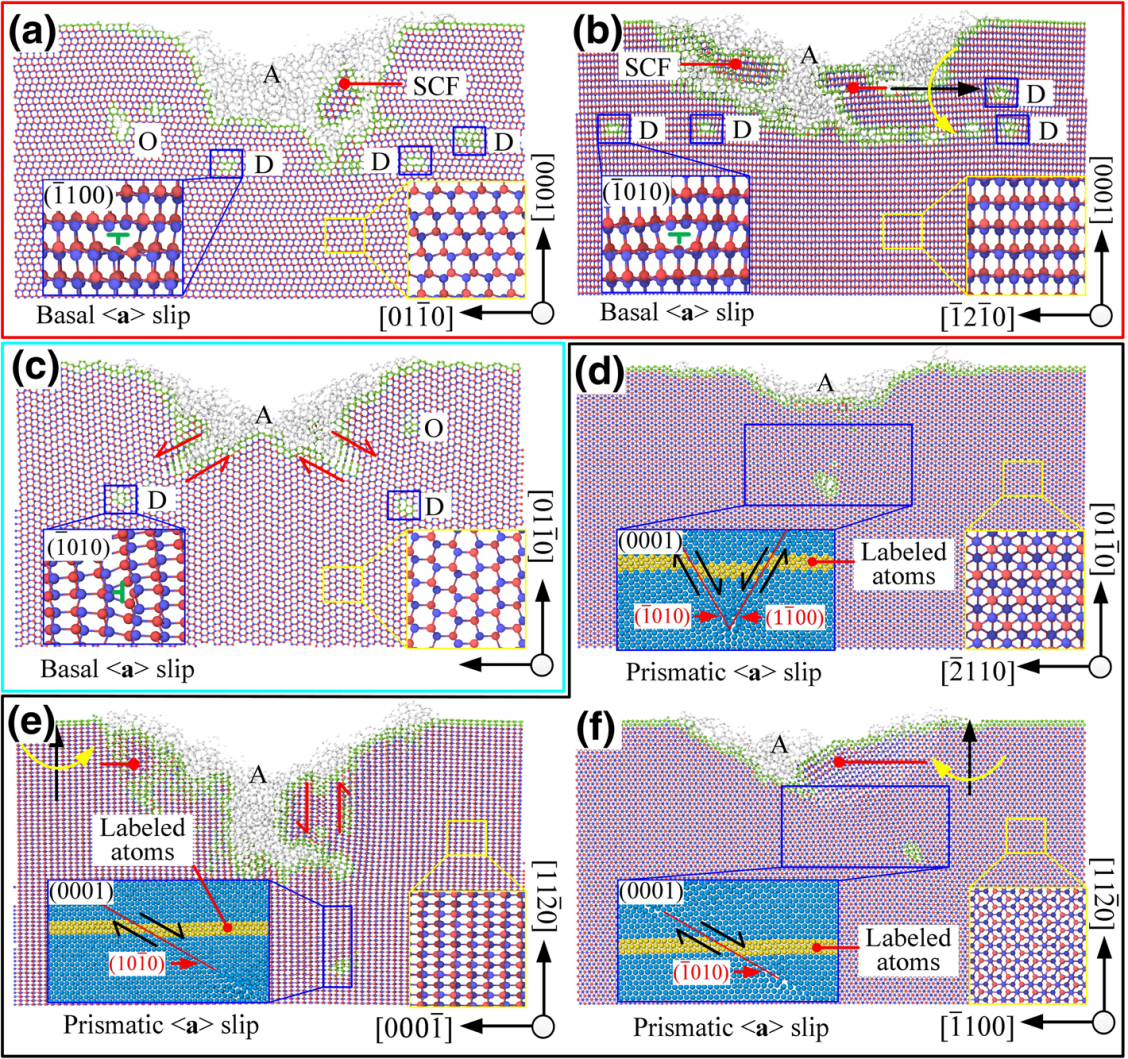
The cross-section of machined area, *D* is dislocation, *A* is amorphous phase, SCF is single-crystal form, *O* is other type of defect, where **a**–**f** are the corresponding process modes $(0001)/\left[2\overline{1}\overline{1}0\right]$, $(0001)/\left[10\overline{1}0\right]$, $\left(01\overline{1}0\right)/\left[2\overline{1}\overline{1}0\right]$, $\left(01\overline{1}0\right)/\left[0001\right]$, $\left(11\overline{2}0\right)/\left[1\overline{1}00\right]$, and $\left(11\overline{2}0\right)/\left[0001\right]$, respectively.

As shown in Fig. 6a, b when $(0001)/\left[2\overline{1}\overline{1}0\right]$ and $(0001)/\left[01\overline{1}0\right]$ were selected, the slip motion mainly occurred on the slip system $(0001)/<1\overline{2}10>$, which is due to pushing forward of the tool. The corresponding sliding movement mode is consistent with the calculation results of the Schmidt coefficient. There were nanocrystalline grains and lattice deflections in the subsurface area and these damages formed some irregular lattice distortion zones. The amorphous phase covered the entire machined surface and the depth of the dislocation was close to the depth of the lattice distortion layer.

When $\left(01\overline{1}0\right)/\left[2\overline{1}\overline{1}0\right]$ is selected, as the Schmidt coefficient of basal <**a**> slip is equal to 0, the basal <**a**> slip should not occur in theory. But the angle between the loading direction $\overset{\rightharpoonup }{f_1}$ and slip direction $\left[11\overline{2}0\right]$ was only 5.3°. Under the shear action due to the relative motion between the contact area and non-contact area (see Fig. 6c), the basal <**a**> slip was triggered before the prismatic slip under the processing mode of $\left(01\overline{1}0\right)/\left[2\overline{1}\overline{1}0\right]$ and occurred on both sides of the V-groove. Under the processing mode of $\left(11\overline{2}0\right)/\left[1\overline{1}00\right]$, the angles between the loading direction $\overset{\rightharpoonup }{f_1}$ and the sliding directions $\left[1\overline{2}10\right]$ and $\left[\overline{1}\overline{1}20\right]$ were 24.7° and 35.3°, respectively. The shearing action did not induce sliding motion of the basal plane. The corresponding slip motion mode is consistent with the calculation results of the Schmidt coefficient. As shown in Fig. 6e, when $\left(11\overline{2}0\right)/\left[1\overline{1}00\right]$ was selected, the slip system $\left[1\overline{2}10\right]/\left(\overline{1}010\right)$ had a high Schmidt coefficient under the joint action of $\overset{\rightharpoonup }{f_1}$ and $\overset{\rightharpoonup }{f_2}$, which would inevitably result in a great depth of the subsurface damage layer. The lattice distortion and the amorphous phase also existed, but unlike machining on the basal plane, dislocation depth was significantly greater than that of the lattice distortion. The *sp*^*3*^→*sp*^*2*^ transition behavior occurred in the subsurface region.

When the processing direction was parallel to the *c*-axis, due to severe wear of tip, prismatic slip caused by the downward extrusion was the primary slip motion mode in the stable scratching stage. The corresponding slip motion mode was consistent with the calculation results of the Schmidt coefficient. When $\left(01\overline{1}0\right)/\left[0001\right]$ was selected, with the loading direction $\overset{\rightharpoonup }{f_2}$, the slip systems $\left[1\overline{2}10\right]/\left(\overline{1}010\right)$ and $\left[\overline{1}\overline{1}20\right]/\left(1\overline{1}00\right)$ had the same Schmidt coefficient, cross-slip occurred on the two slip surfaces with an angle of 60°, and then, the pinning effect occurred which hindered the slip motion. Thus, the maximum subsurface damage depth *SSD*_max_ would be less than or equal to *h*_*t*_ tan *θ*/2 cot *α*/2, where *θ* = 101° is the theoretical angle of the nanogroove and α = 60° is the angle between the slip plane $\left(\overline{1}010\right)$ and $\left(1\overline{1}00\right)$. When $\left(11\overline{2}0\right)/\left[0001\right]$ was selected, the slip systems $\left[\overline{2}110\right]/\left(0\overline{1}10\right)$ and $\left[1\overline{2}10\right]/\left(\overline{1}010\right)$ also had the same Schmidt coefficient, but the slip motion was affected by the irregular wear of the tip and only the slip system $\left[1\overline{2}10\right]/\left(\overline{1}010\right)$ occurred.

**Fig. 7:**
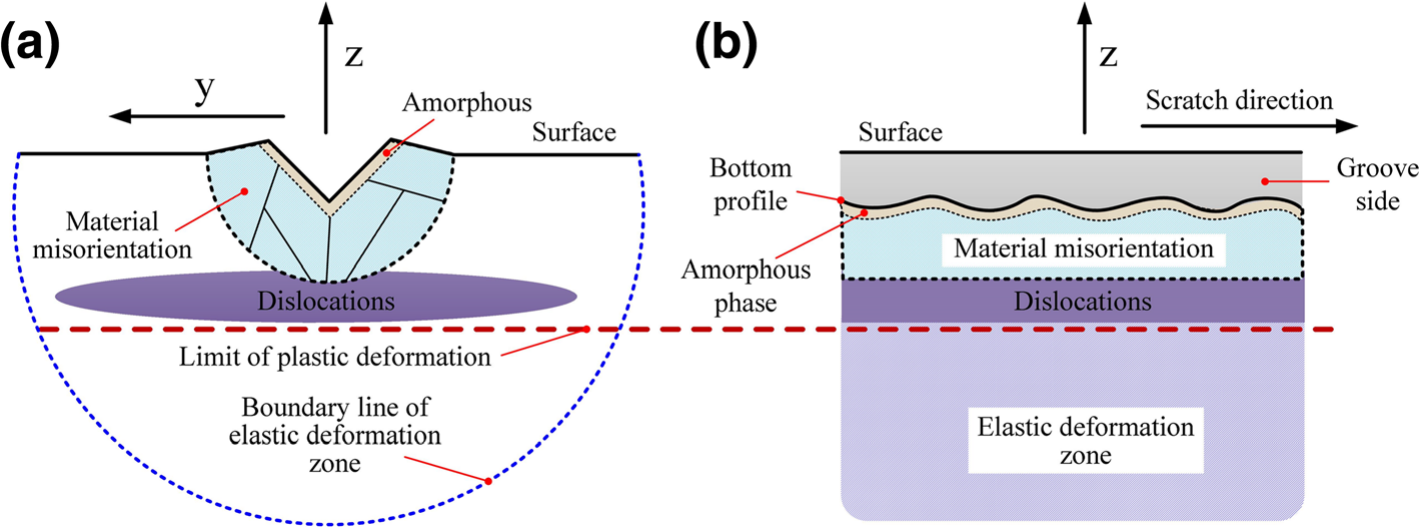
Processed surface/subsurface damage form distribution. **a** *xy* cross-section. **b** *xz* cross-section

In summary, as shown in Fig. 7, the damages in the subsurface regions under different processing conditions were primarily dislocations, lattice distortion (torsion/relative sliding), and amorphous phase. The deformation of 6H-SiC was mainly caused by the slip motion, non-crystallization of materials, and irregular lattice distortion. The main slip deformation modes were basal slip and prismatic slip, which are closely related to the processing surface/direction.

## Concluding Remarks

In this work, the deformation mechanism and nanometric machinability of 6H-SiC were investigated under different combinations of crystal plane (machining surface)/crystal orientation (machining direction) and the following conclusions are drawn:

1. The deformation mechanism of 6H-SiC during the scratching process at the nanoscale is mainly the result of the combination of amorphous phase transition, lattice distortion, and dislocation slip motion. The depth of the dislocation line determines the subsurface damage depth in the machined area of 6H-SiC.
2. Basal <**a**> slip and prismatic <**a**> slip motion plays a dominant role in the slip deformation of 6H-SiC during scratching process. In addition to the processing mode prismatic plane/<**a**>, the slip motion during the scratching process of 6H-SiC could be predicted via Schmidt algorithm.
3. The processing mode $\left(01\overline{1}0\right)/\left[2\overline{1}\overline{1}0\right]$ is conducive for achieving a high removal rate and low abrasive wear, which is apt for machining of the 6H-SiC surface. The basal plane and *c*-axis are the difficult-to-machine face and direction of 6H-SiC, which can be used as a reference for the design of cutting tool.

## Data Availability

All data generated or analyzed during this study are included in this article.

## Abbreviations

*A*: Amorphous phase
ABOP: Analytical bond order potential
*D*: Dislocation
*d*: Undeformed chip thickness
GSF: Generalized stacking fault energy
LAMMPS: Large-scale atomic/molecular massively parallel simulator
MD: Molecular dynamics
MOSFET: Metal-oxide-semiconductor field-effect transistor
NVE: Number, volume, and energy
*O*: Other type of defect
SCF: Single-crystal form
SPDT: Single-point diamond turning
SSD: Subsurface damage depth
SSD_max_: Maximum subsurface damage depth
